# Electrotaxis-on-Chip to Quantify Neutrophil Migration Towards Electrochemical Gradients

**DOI:** 10.3389/fimmu.2021.674727

**Published:** 2021-08-06

**Authors:** Maryam Moarefian, Rafael V. Davalos, Michael D. Burton, Caroline N. Jones

**Affiliations:** ^1^Department of Biological Sciences, Virginia Tech, Blacksburg, VA, United States; ^2^Department of Biomedical Engineering and Mechanics, Virginia Tech, Blacksburg, VA, United States; ^3^Department of Neuroscience, Neuroimmunology and Behavior Group, School of Behavioral and Brain Sciences, University of Texas at Dallas, Richardson, TX, United States; ^4^Department of Bioengineering, University of Texas at Dallas, Richardson, TX, United States

**Keywords:** electrotaxis, neutrophil, migration, microfluidics, wound healing, immunomodulation

## Abstract

Electric fields are generated *in vivo* in a variety of physiologic and pathologic settings, including wound healing and immune response to injuries to epithelial barriers (e.g. lung pneumocytes). Immune cells are known to migrate towards both chemical (chemotaxis), physical (mechanotaxis) and electric stimuli (electrotaxis). Electrotaxis is the guided migration of cells along electric fields, and has previously been reported in T-cells and cancer cells. However, there remains a need for engineering tools with high spatial and temporal resolution to quantify EF guided migration. Here we report the development of an electrotaxis-on-chip (ETOC) platform that enables the quantification of dHL-60 cell, a model neutrophil-like cell line, migration toward both electrical and chemoattractant gradients. Neutrophils are the most abundant white blood cells and set the stage for the magnitude of the immune response. Therefore, developing engineering tools to direct neutrophil migration patterns has applications in both infectious disease and inflammatory disorders. The ETOC developed in this study has embedded electrodes and four migration zones connected to a central cell-loading chamber with migration channels [10 µm X 10 µm]. This device enables both parallel and competing chemoattractant and electric fields. We use our novel ETOC platform to investigate dHL-60 cell migration in three biologically relevant conditions: 1) in a DC electric field; 2) parallel chemical gradient and electric fields; and 3) perpendicular chemical gradient and electric field. In this study we used differentiated leukemia cancer cells (dHL60 cells), an accepted model for human peripheral blood neutrophils. We first quantified effects of electric field intensities (0.4V/cm-1V/cm) on dHL-60 cell electrotaxis. Our results show optimal migration at 0.6 V/cm. In the second scenario, we tested whether it was possible to increase dHL-60 cell migration to a bacterial signal [N-formylated peptides (fMLP)] by adding a parallel electric field. Our results show that there was significant increase (6-fold increase) in dHL60 migration toward fMLP and cathode of DC electric field (0.6V/cm, n=4, p-value<0.005) *vs*. fMLP alone. Finally, we evaluated whether we could decrease or re-direct dHL-60 cell migration away from an inflammatory signal [leukotriene B_4_ (LTB_4_)]. The perpendicular electric field significantly decreased migration (2.9-fold decrease) of dHL60s toward LTB_4_ *vs*. LTB_4_ alone. Our microfluidic device enabled us to quantify single-cell electrotaxis velocity (7.9 µm/min ± 3.6). The magnitude and direction of the electric field can be more precisely and quickly changed than most other guidance cues such as chemical cues in clinical investigation. A better understanding of EF guided cell migration will enable the development of new EF-based treatments to precisely direct immune cell migration for wound care, infection, and other inflammatory disorders.

## Introduction

Cell migration plays a critical role in biological processes such as immune response to infection, wound healing (1–3), cell isolation, cell separation (4, 5), cancer metastasis (6–9), and immune-inflammatory responses (10). Electrotaxis is the cell directional movement under the effect of electric fields. The term -taxis was used by researchers to indicate the gradient of physical or chemical cues. Chemical gradients in tissue cause a chemical stimulus (11) for cell migration (chemotaxis). The observation that cells can follow chemoattractant was first studied by scientists who discovered the mechanisms underlying the attraction of neutrophils to the sites of infection (11). However, directional cell movements toward attractive and away from repulsive signals are notably critical in almost all physiological processes. Physiological and externally applied electric fields (electrotaxis/galvanotaxis) are essential factors for inducing cell migration in the tissue microenvironment. Electrotaxis is a guiding mechanism for the orientation and directional movements of many cell types, including fission yeast cells (12), *Caenorhabditis elegans* (13–16), pathogenic bacterium such as *Pseudomonas aeruginosa*, *Escherichia coli*, and Dictyostelium (17–20). It has been reported that some cells migrate toward the cathode [e.g., neural stem cells (21–26), fibroblasts (27–29), keratinocytes (30–33), rat prostate cancer cells (34, 35), T lymphocyte (36–40), lung cancer cells (41–45), and many epithelial cell types (3, 30, 46–51)], and other cells migrate to the anode [e.g., corneal endothelial cells (52), breast cancer cells (53, 54), glioblastoma (55, 56), and human vascular endothelial cells (57, 58)]. Signaling pathways involved in the electrotaxis phenomenon are still not fully understood. Recent studies demonstrate electromigration (electrophoretic and electroosmotic) of cell surface receptors, voltage-gated ion channels in cells for calcium signaling (59), and asymmetric ion distribution and electrotaxis of ions inside the cell are some cellular mechanism of sensing and responding to cellular electric fields (60). The directional movement of cells is due to an electrostatic polarity associated with cellular structure and cell-cell/cell-ECM interaction. Developmental polarity is observed along three axes, anterior-posterior, dorsal-ventral, and left-right in biological cells. Such polarities can be established by concentration gradients of secreted proteins and asymmetric organization of cellular components, such as the cytoskeleton (28, 61).

Investigation of the effects of electric fields on biological cells in polymer-based microfluidics has been the interest of many researchers over two decades (62–64). Many of these studies focused on separation, sorting, and isolation of cells (4, 5). However, studying the active electrotaxis of cells with high temporal-resolution is important in understanding immune cell trafficking behaviors in the human body (65). The migration of neutrophils toward the cathode of an electric field was previously reported with endogenously generating electrical gradient (66, 67). In the previous groundbreaking work (66), neutrophils migrated alongside epithelial cells guided by an electric field modeling wound healing. The study also mapped individual mouse neutrophil migratory trajectories toward the cathode of an electric field on a planar surface. Peretz-Soroka et al. developed a model to predict the electro-mechano-chemical coupling, where free energy ATP hydrolysis is transformed in the power of electrically polarized cell movement. In this study, they demonstrated that cells pre-stimulated by fMLP electrically-polarized and spread out to form a planar migratory mode and demonstrated a memory effect of cells migrating for up to 10 min after EF was turned off (67). In the current investigation, we quantified the significance of neutrophil-like cells migration toward the cathode in the presence of a defined chemoattractant gradient. In our novel ETOC platform the electric field and chemoattractant concentrations can be precisely controlled and neutrophil directional migration can be easily tracked in channels. Our ETOC platform allowed us to optimize electric field conditions in the presence of a controlled chemoattractant gradient and required less cells and reagents.

In this work, we developed a new electrotaxis-on-chip (ETOC) platform to explore the potential of electric fields in driving neutrophils towards an infection or away from an inflammatory microenvironment. To better understand the *in vivo* complexity of neutrophil migratory decision-making, it is essential to recapitulate chemoattractant and electric field conditions more accurately using an *in vitro* experimental model. Measuring individual cell velocity and directionality *in vivo* requires precise control of the tissue spatiotemporal microenvironment. Microfluidic chemotaxis assays have been shown to assist researchers to address neutrophil migration under spatiotemporally controlled chemical gradients (68–72). Also, engineering a novel ETOC platform has various advantages such as reduction of joule heating, facilitation of high through-put investigation, and precise control of electric fields, cells, and reagents (73). Dual gradient microfluidic platforms have been used by our group and other researchers to study neutrophil chemotaxis with coexisting pro-inflammatory and chemotactic signals mimicking those released by tissue bacteria (74–77). Researchers have also previously investigated the effect of co-existing chemotaxis and electrotaxis on cell migration (39). Lymphocyte chemotaxis (78), electrotaxis (36, 37), and co-existing chemotaxis and electrotaxis (40) show the migration of T-cells toward the cathode. The study of T-cells migration suggested greater electrotactic attraction of T-cells toward cathode of DC electric fields in the presence of a competing CCL19 chemoattractant gradient. However, a microfluidic device for quantifying neutrophil time-dependent migration pattern and decision making with co-existing electrotaxis and chemoattractants [pro-inflammatory (LTB_4_) and chemotactic (fMLP)] has not been previously investigated.

**Figure 1:**
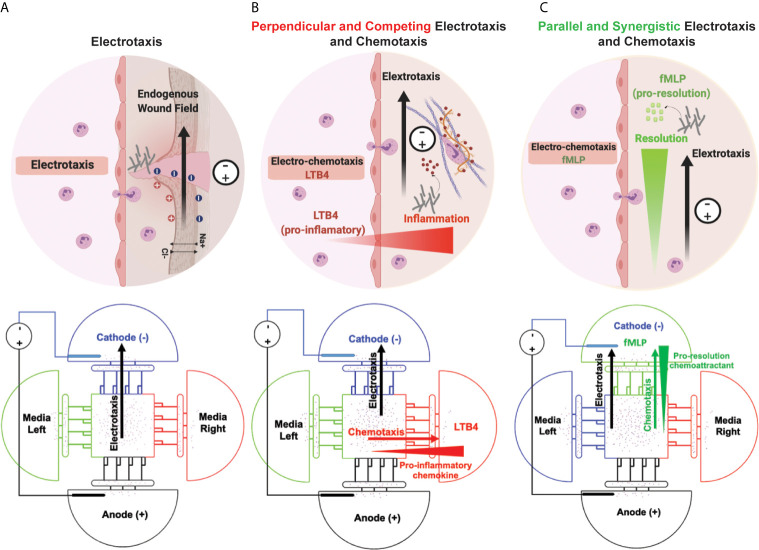
Simultaneous neutrophil chemotaxis and electro taxis in a microfluidics platform. **(A)** Electrotaxis of neutrophils toward wounds’ endogenous electric fields and externally applied electric fields. The schematic of the microfluidic experiment design for investigating the effect of electro taxis signal on neutrophil migration in the absence of chemoattractant. **(B)** Decision-making of neutrophils towards an inflammatory chemoattractant (LTB_4_) and perpendicular electric field. The schematic of the microfluidic experiment design for investigating the effect of pro-inflammatory electrotaxis and chemotaxis signals on neutrophil migration. **(C)** Neutrophil electrotaxis towards an infection with parallel chemoattractant (fMLP) and electric field. The schematic of the microfluidic experiment design for investigating the effect of electrotaxis and chemotaxis signals on neutrophil migration.

We have previously reported on iontophoretic drug delivery in a microfluidic device and will now apply this same concept to drive immune cell migration (79). Neutrophils are the immune system’s first responders against pathogenic infection after chronic wounds and injuries and linking innate and adaptive immunity in inflammatory immune responses. Endogenous DC electric field (dcEF) plays an important role in wound healing (3, 49, 80), tissue regeneration (59, 81, 82), and embryogenesis (83). In addition to chemical stimuli (neutrophil-like cells chemoattractant), the endogenous DC electric field may influence neutrophil-like cells migration to the infectious (**Figure 1A**). In this study, we designed and validated a novel four-sided microfluidic ETOC platform for studying neutrophil-like cells migratory decision-making toward fMLP or LTB_4_ in the presence of an electric fields. Recent microfluidics electrotaxis assays (30, 36, 37, 39, 40, 43, 45, 48, 73, 84, 85) used electric field intensity between (4V-20V) to reach the target of 0.4V/cm-4V/cm electric field for inducing cell electrotaxis in microchannels. The endogenous electric field experimentally measured in wound healing was 0.4V/cm-2V/cm, and many clinical trials reported a significant increase in the rate of wound healing from 13% to 50% (86). However, exogenous electric fields of higher intensities used for transdermal drug delivery (87), increase the permeability of cell membrane (88), and a therapeutic tool for restoring tissue integrity in severe injuries with the exogenous electric field of less than 4 V/cm. The electric field can synergistically drive a higher percentage of neutrophils toward a chemoattractant (fMLP) signal or reduce the number of neutrophils migrating toward an inflammatory signal (LTB_4_). We were able to direct neutrophils away from pro-inflammatory signals (LTB_4_) (perpendicular field) (**Figure 1B**), as well as increase neutrophil-like cells migrating towards fMLP (parallel field) (**Figure 1C**). LTB_4_ and fMLP induce a respiratory burst in human neutrophils (89). N-Formyl-Met-Leu-Phe (fMLP), a mimic of N-formyl oligopeptides that are released from bacteria, is a potent neutrophil chemoattractant at the site of infection. Also, fMLP induces cytokines (e.g., TNFα) release by macrophages in microbial infection, which is the cause of self-limited tissue barrier against the inflammatory response of neutrophils (90, 91) (**Figure 1**). We hypothesize the effect of exogenous and endogenous electric fields for enhancing the chemotactic effect of fMLP and facilitating the migratory pathway for neutrophils (**Figure 1C**). On the other hand, LTB_4_ is the lipid leukotriene B_4_ and the pro-inflammatory pathway for neutrophils, which cause the neutrophil inflammation in healthy tissue such as skin and lungs. Neutrophil accumulation in the lungs causes damage to healthy endothelial and epithelial cells. It would be beneficial to redirect neutrophils in this hyperinflammatory state toward controlled electrotaxis signals. Neutrophil migration induced by the externally applied electric field may enable the reduction of neutrophil migration towards an inflammatory chemoattractant (LTB_4_) (**Figure 1B**). In the future, EF-based treatments may be used to precisely direct immune cell migration for inflammatory disorders.

## Materials and Methods

### Device Design and Fabrication

**Figure 2:**
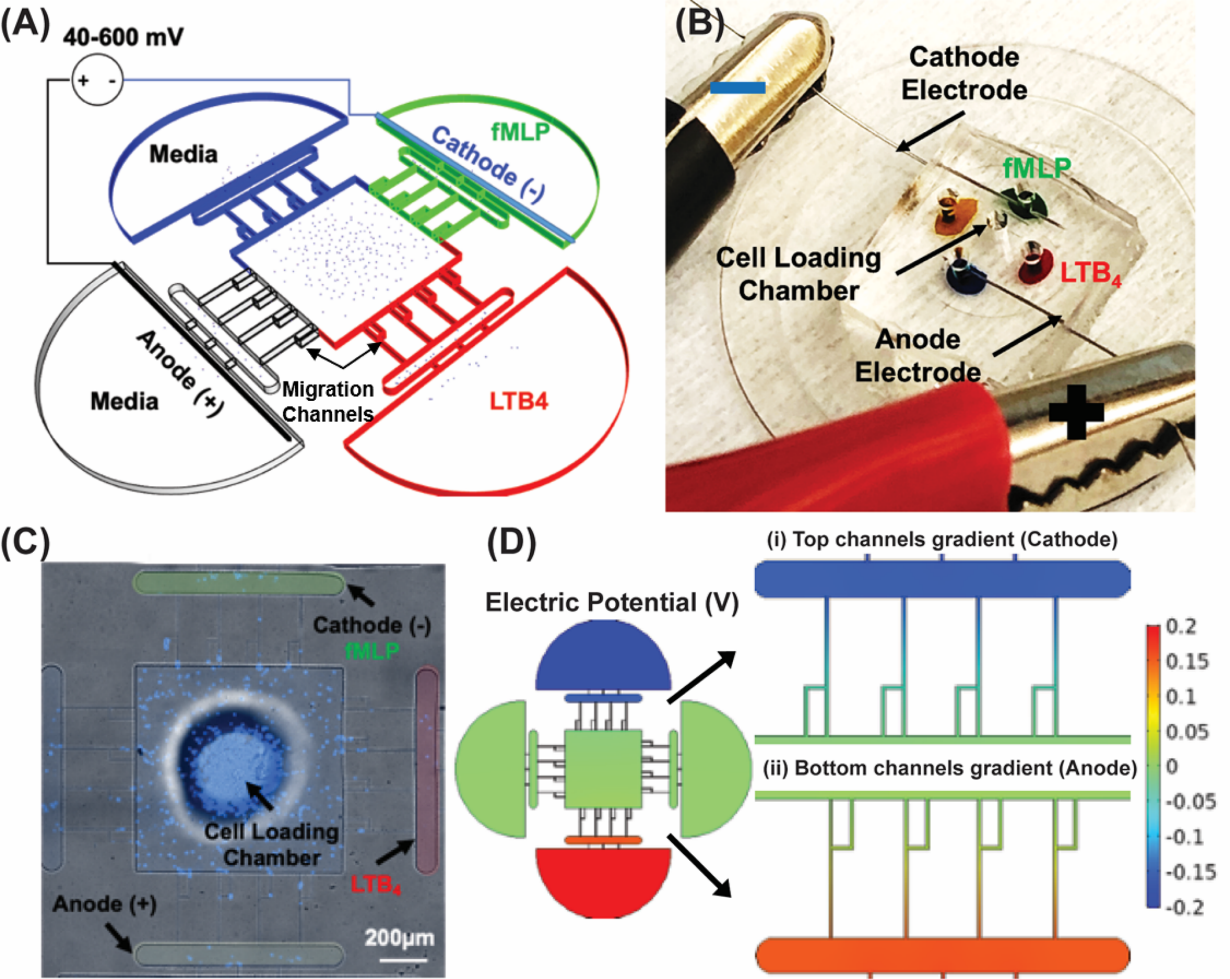
Electrotaxis-On-Chip (ETOC) microfluidic platform. **(A)** Microfluidic device design. Control reservoir (blue) contains a complete cell medium. fMLP chemoattractant reservoir (green). LTB_4_ chemoattractant reservoir (red). Anode reservoir (brightfield) contains a complete cell medium. Central cell-loading chamber for loading neutrophil. Four linear migration channels connecting the central cell-loading to reservoirs for quantifying neutrophil electrotaxis. **(B)** PDMS based microfluidic device, LTB_4_ chamber color-coded with a red food dye and fMLP chamber color-coded with a green food dye. The anode chamber is blue, and the media chamber is orange color-coded. Stainless steel electrodes were inserted in the cathode and the anode chamber. The cell loading chamber is at the center of the device. **(C)** Nikon TiE microscope 10X image of the microfluidic device and stained dHL60 by Hoechst stain (DAPI fluorescent DNA stains), scale bar 200 µm. **(D)** The electric potential in the migration channels. (i) Cathodic channels. (ii) Anodic channels.

A microfluidic competitive chemotaxis chip (μC3) previously reported in our study (77, 92) is designed with two chemoattractant reservoirs that enable the formation of a chemoattractant gradient. The adopted design [Electrotaxis-on-Chip (ETOC)] includes electrodes to precisely control electric fields in the cell migration channels. We have previously reported on incorporating electrodes into microfluidic platforms to model iontophoretic drug delivery and use similar methods in this study (79). The ETOC device consists of four parts: (i) Control reservoir (blue) contains the complete cell medium. (ii) fMLP chemoattractant reservoir (green). (iii) LTB_4_ chemoattractant reservoir (red). (iv) Anode reservoir (black) contains the complete cell medium. (v) Central cell-loading chamber for loading neutrophil-like cells. (vi) Four linear migration channels connecting the central cell-loading to reservoirs for quantifying neutrophil-like cells electrotaxis (**Figure 2A**). **Figure 2B** demonstrates the device and experimental design. The TRANS and DAPI images of the microfluidic device were taken by Nikon TiE microscope with 10X objective, and stained dHL60 by Hochstein (DAPI fluorescent DNA stains) were loaded inside the device (**Figure 2C**). The COMSOL simulations showed electric potential (**Figure 2D**) in the migration channels.

A microfluidic device for quantifying neutrophil-like cells migration pattern was designed and fabricated using standard photolithography techniques (93). Two standard photolithography techniques were used to create a silicon mold from two separate masks, chemoattractant wells, and migration channels. Mask aligner (Karl Suss MA-6 Mask Aligner) was used to align two separate masks. Replication molding techniques facilitate the fabrication of PDMS (polydimethylsiloxane) microfluidic device (94). Mixing the PDMS and curing agent with a 10:1 weight ratio prepared PDMS pre-polymer (Sylgard 184; Dow Corning, Waltham, MA). The PDMS prepolymer was then degassed in the desiccator and poured onto prepared silicon mold. The PDMS was cured at 65°C for 8hr. After curing, the inlets and outlets were punched using a 0.75 mm biopsy puncher. Finally, PDMS device was bonded to a glass slide using nitrogen plasma bonding [Nordson MARCH (AP-300)] for mechanical stability and place on an 80°C hot plate for 45 min.

### Cell Preparation and Loading

Human promyelocytic leukemia cells (HL60 CCL-240, American Type Culture Collection ATCC, Manassas, VA) were used in this study. Iscove’s Modified Dulbecco’s Medium (IMDM, ATCC, Manassas, VA) supplemented with 10% fetal bovine serum (FBS, ATCC, Manassas, VA) were used as a complete media for HL60 cells. Cells were cultured (90% confluent) in complete media and incubated at 37°C in 5% CO_2_. 1.5% Dimethyl sulfoxide (DMSO, Sigma-Aldrich, St. Louis, MO) was added to 1.5X10^6^ cells. mL^−1^ of HL60s and incubate for 4-5 days to differentiate cells to a neutrophil-like state (denoted as dHL60 cells) following ATCC culture guidelines and protocols previously established in our laboratory (77). dHL60s were spun down (130G) at RT for 7 min before the experiment and resuspended in fresh media. Then, the central cell-loading chamber for loading neutrophil-like cells (**Figure 2A**) was filled by dHL60 cells (400,000 cell/40 μL) using a gel loading pipette tip. Devices were washed with 1X PBS (Thermo Fisher Inc.) twice; then, plates were filled with complete media before the experiment. Complete media were changed before time-lapse imaging. Viability of dHL60 cells loaded into the microfluidic platform was >90% viable, as confirmed by live and dead cell staining assay after 8 hours of time-lapse imaging with 600 mV and without chemotaxis assay (**Supplementary Figure 1**).

### Electrotaxis Assay and Experiment Setup

Complete media (IMDM+10% FBS) salt bridge was used to connect electrotaxis wells in the microfluidic device. Sterile stainless-steel acupuncture needles (Kingi, China) with a diameter of 0.12 mm were placed at the inlet and outlet of the electrotaxis wells to deliver DC electric field, and stainless-steel wires (Zoro, Inc.) were used to construct electric field circuit. Electrodes were fixed using Epoxy glue (Devcon Inc.), and devices were washed stay in 1X PBS (Thermo Fisher Inc.) for 30 min twice and washed twice for removing any toxicity from Epoxy glue. Optimal electric field intensities were chosen to induce maximal dHL60 cell migration for both endogenous DC field and applied DC field. The characteristic length of the current microfluidic device is 0.15 cm. Therefore, we chose an applied voltage from 0 mV to 600 mV to examine reported electric field intensities in clinical and *in vivo* investigations (0.4 V/cm-4 V/cm). The electrotaxis conditions include 1. An endogenous potential field modeling wound healing (<100 mV). 2. External applied electric potential (<600mV). The intensity of the DC electric field is very low and it is not high enough for the generation of electrolysis and bubble generation during the experiment. Also, the media on top of the microfluidics device has changed every four hours for maintaining the same level and prevent acidification. The phenol red color of the media did not change significantly during the experiment.

### Chemotaxis Assay

Fibronectin is a large and the most abundant glycoprotein in the extracellular matrix (92). In has been used in previous microfluidic-based studies for increasing cell adhesion (6). Microfluidic channels were coated using 50 μL fibronectin (Sigma-Aldrich, St. Louis, MO) [10 μg/mL] to mimic the extracellular matrix (ECM) neutrophil-like cells adhesion promotion. After adding fibronectin on top of the device, the device was then placed in a vacuum desiccator for 10 min and an additional 45 min to 1 hour at the room temperature for fibronectin adsorption to the glass and PDMS channel surfaces. The drop of fibronectin should cover all punches to let the air displaced by fibronectin solution in PDMS channels. The 6-well plates were filled with 4.5 ml of 1X PBS. Chemoattractants Leukotriene B4 (LTB_4_, Cayman Chemical, Ann Arbor, MI) and (N-Formylmethionine-leucyl-phenylalanine (fMLP, Sigma-Aldrich, St. Louis, MO) were diluted using complete media (IMDM+10%FBS). Ten microliters of each chemoattractant solution (fMLP, [10 nM] and LTB_4_, [100 nM]) were then loaded into the chemoattractant reservoirs. The first set of experiments are without chemoattractant. In the second set, LTB_4_ chemoattractant was loaded using gel loading pipettes. The third set of experiments was with fMLP chemoattractant. Clinically relevant optimal chemoattractant concentrations previously reported (6) for inducing maximal dHL60s migration.

### Live Microscopy and Image Processing

Nikon TiE fully-automated microscope equipped with a Plan Fluor 10x Ph1 DLL (NA = 0.3) lens and 37°C with 5% carbon dioxide incubator was used for time-lapse imaging experiments. NIS-elements (Nikon Inc., Melville, NY) software facilitates image capturing and analysis conducted by using ImageJ. Images were recorded using a bright-field channel at six-minute intervals for 8 hr. Live/dead images were captured using FITC (green) and TRITC (red) fluorescent channels. The number of cells per channel migrating toward chemoattractant, cathode, and anode reservoirs, was quantified as followed: (1) Control (no potential) (2) Electrotaxis (3) Co-existing chemotaxis and electrotaxis. We used dHL60s cell type as neutrophils. DC electric potential variations are: 0 mV, 40 mV, 80mV, 200 mV, 400 mV, and 600 mV. DC electric field variation are: 0 V/cm, 0.27 V/cm, 0.53 V/cm, 1.33 V/cm, 2.67 V/cm, and 4 V/cm. Low-intensity DC potentials (0 mV, 40 mV, and 80mV) mimic endogenous DC fields, and high-intensity potentials (200 mV, 400 mV, and 600 mV) mimic applied DC fields. We used two chemoattractants at optimal concentrations to induce dHL60 chemotaxis: (1) LTB_4_ (pro-inflammatory): [100 nM]. (2) fMLP (chemotactic): [10 nM].

### Statistical Analysis

Prism version 8.1.2 (332) software (GraphPad Software, La Jolla, CA) with a confidence level of α = 0.05 was used for statistical analyses. Pair t-test comparison was used for comparing a control condition to electric field conditions (n=4) as well as dHL60 viability in the microfluidic device after 8 hours of migration experiment (n=2). Data are presented as arithmetic mean ± SD. “n” represent the number of biological samples.

## Results

### Effect of DC Electric Potential on dHL60’s Electrotaxis

**Figure 3:**
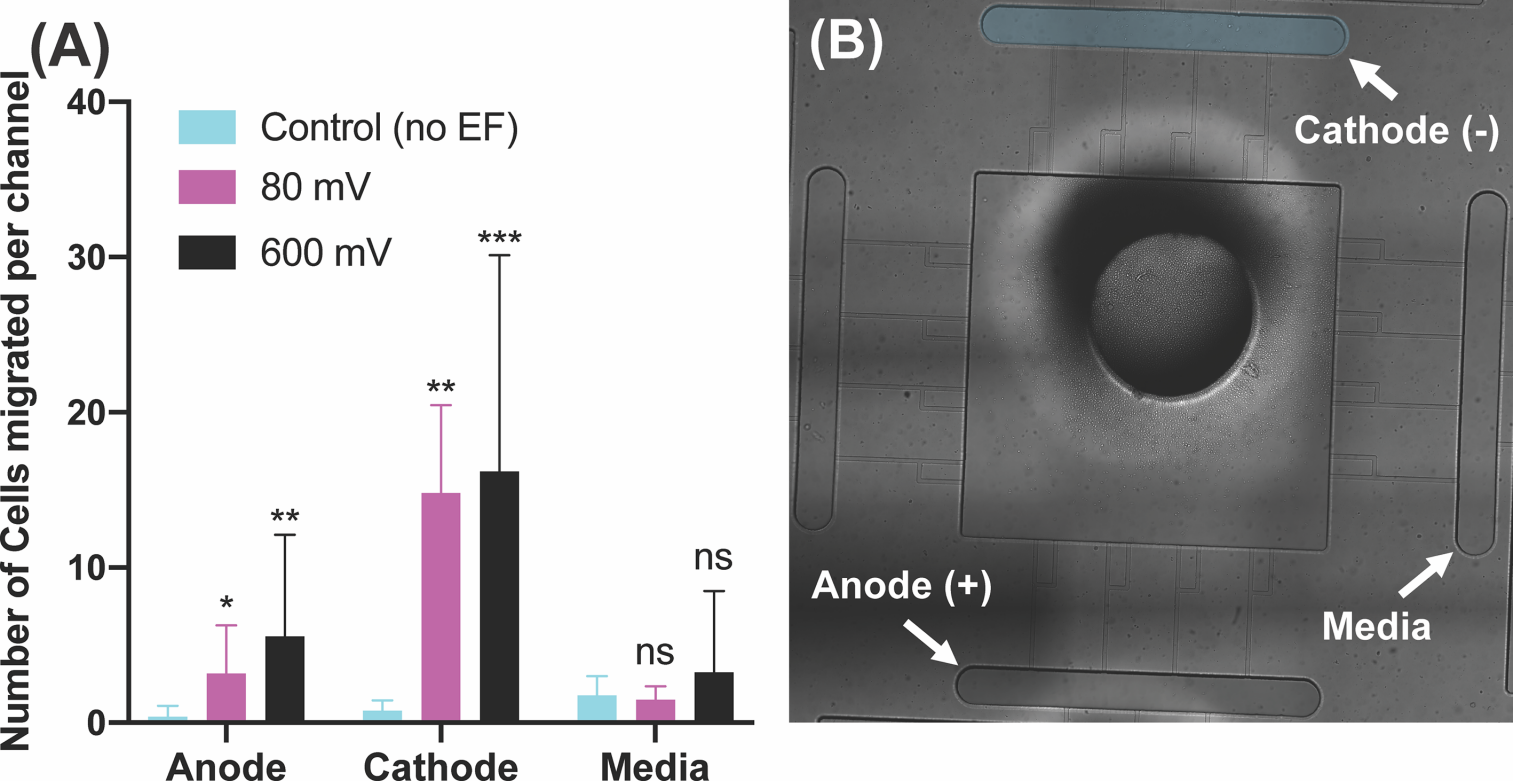
Neutrophil-like cells electrotaxis under the effect of the DC electric field. **(A)** Quantification of the number of neutrophils migrated per channel toward the cathode. The result shows a significant increase (65%-80%) in migration by applying different DC electric field strength (n=4, p-value<0.005). Quantification of the number of neutrophils migrated per channel toward the anode. The result indicates a significantly low, less than ~5 cells per channel, directional movement of neutrophils toward the anode. Quantification of the number of neutrophils migrated per channel toward the complete media. The result shows a significant low, less than ~3 cells per channel, migration toward the complete media due to no electrical or chemical signals. **(B)** Nikon TiE microscope 10X image of the microfluidic device and experiment setup of electrotaxis. *P ≤ 0.05; **P ≤ 0.01; ***P ≤ 0.001; ns, not statistically significant.

We inspected the effect of electric fields on the dHL60 cells (neutrophil-like cells) loaded in the central chamber of our electrotaxis-on-chip (ETOC) platform. Five different potential intensities were investigated in this section to simulate endogenously (<100 mV) and externally applied potentials (<600mV). In the absence of an electric field (denoted as EF), dHL60s had a significantly low migration, less than ~5 cells per channel, into the four side-chambers. After electric field stimulation, neutrophils migrated toward the cathode with an order-of magnitude increase in numbers. Examining the effect of externally endogenous potential (<100mV) intensities showed a significant migration of neutrophils toward cathode at 40mV (n=4, p-value=0.0006) and 80mV (n=4, p-value=0.0026). Also, 40mV applied potential indicated more significant migration than 80mV. On the other hand, 600mV (n=4, p-value=0.0003) indicated the most significant migration toward the cathode in the range of externally applied potential (**Figure 3A** and **Supplementary Video 1**). Migration toward media was significantly low, less than ~5 neutrophils per channel, which is expected due to the elimination of electrical signal and chemical stimuli (**Figure 3**). The most critical finding of neutrophil-like cells directional movement during electrotaxis is the low migration of neutrophils toward the anode (**Supplementary Video 2**), less than ~5 neutrophils per channel, and significant migration toward the cathode, with 20-30 cells per channel (n=4, p-value<0.005), which indicated the neutrophils’ positive polarity. The electric potential spectrum for the first scenario, only electrotaxis, has been shown in **Supplementary Figure 2**.

### Effect of DC Electric Potentials Co-Exist With LTB_4_ Chemoattractant on dHL60 Migration

**Figure 4:**
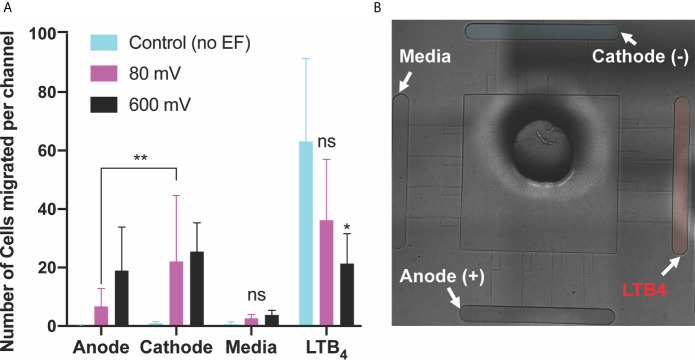
Neutrophils-like cells electrotaxis under the effect of competing pro-inflammatory chemoattractant gradient and DC electric field. **(A)** Quantification of the number of neutrophils migrated per channel toward the anode. The result indicates a significantly low, less than ~5 cells per channel, directional movement of neutrophils toward the anode. Neutrophil-like cells migration toward the anode is around 50% more than neutrophil-like cells migration toward a complete media. Quantification of the number of neutrophils migrated per channel toward the pro-inflammatory chemoattractant gradient (LTB_4_). Results show a significant decrease (60%-70%) in neutrophil-like cells migration toward LTB_4_ by applying external electric potentials (400mV (n=4, p-value=0.0233) and 600 mV (n=4, p-value=0.0325)). Quantification of the number of neutrophils migrated per channel toward the complete media. Results show no neutrophil-like cells migration toward the complete media due to no electrical or chemical signals. Quantification of the number of neutrophils migrated per channel toward the cathode. The result shows a significant increase (80%-90%) in migration by applying different DC electric field strength (n=4, p-value<0.005). **(B)** Nikon TiE microscope 10X image of the microfluidic device and experiment setup of perpendicular and competing electrotaxis and LTB4 chemotaxis. *P ≤ 0.05; **P ≤ 0.01; ns, not statistically significant.

We then examined the effect of co-existing pro-inflammatory chemotaxis (LTB_4_ gradient) and electrotaxis. LTB_4_ [100 nM] was added to one side of the device for generating a perpendicular pro-inflammatory chemoattractant gradient to DC electric fields to test the neutrophil-like cells decision making. The switching direction of neutrophils to LTB_4_ chemoattractants was observed in the second set of the experiment. The perpendicular chemoattractant gradient attenuated neutrophils migration toward the cathode. However, the migration of neutrophils toward the cathode significantly increased by applying electric fields. Neutrophil-like cells migration toward the cathode showed that potentials of 40mV (n=4, p-value=0.0008), 80mV (n=4, p-value<0.0001), 200mV (n=4, p-value=0.0001), 400mV (n=4, p-value<0.0001), and 600mV (n=4, p-value=0.0026) induced a significant migration toward the cathode in the presence of pro-inflammatory chemoattractant gradients in the perpendicular direction (**Figure 4A** and **Supplementary Video 3**). The migration of neutrophils toward LTB_4_ significantly decreased (60%-70%, n=4, p-value<0.005) by applying electric fields under potentials of 400 mV (n=4, p-value=0.0233) and 600 mV (n=4, p-value=0.0325). However, low strength fields (caused by potentials <400 mV) did not attenuate neutrophil-like cells migration toward the cathode (p-value=0.7-0.9) (**Figure 4A**). A similar result to the first set experiment was obtained in neutrophil-like cells migration toward media due to no electrical or chemical cure in the media chamber (**Figure 4**). Migration toward the anode showed a trend of increased migration by increasing applied field. However, neutrophils’ movement toward the anode, less than ~15 cells per channel (not significant). The electric potential spectrum for the second scenario, perpendicular and competing LTB4 and electrotaxis, has been shown in **Supplementary Figure 3**.

### Effect of DC Electric Potentials Co-Exist With fMLP Chemoattractant on dHL60 Migration

**Figure 5:**
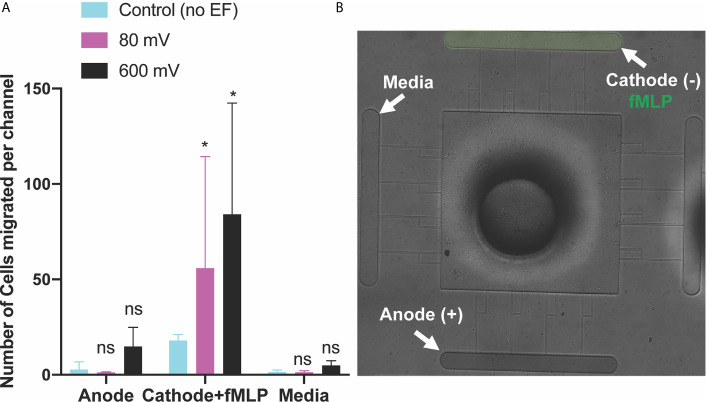
Neutrophils-like cells electrotaxis under the effect of fMLP chemoattractant gradient and DC electric field. **(A)** Quantification of the number of neutrophils migrated per channel toward the cathode and fMLP chemoattractant. The result shows a significant increase (85%-95%) in migration by applying different DC electric field strengths with potentials of 80 mV and 600mV (n=4, p-value<0.001) across the chip. Quantification of the number of neutrophils migrated per channel toward the anode. The result indicates a significantly low, less than ~5-10 cells per channel, directional movement of neutrophils toward the anode. Quantification of the number of neutrophils migrated per channel toward the complete media. Results show no neutrophil-like cells migration toward the complete media due to no electrical or chemical signals. **(B)** Nikon TiE microscope10X image of the microfluidic device and experiment setup of parallel and synergistic electrotaxis and fMLP chemotaxis. *P ≤ 0.05; ns, not statistically significant.

**Figure 6:**
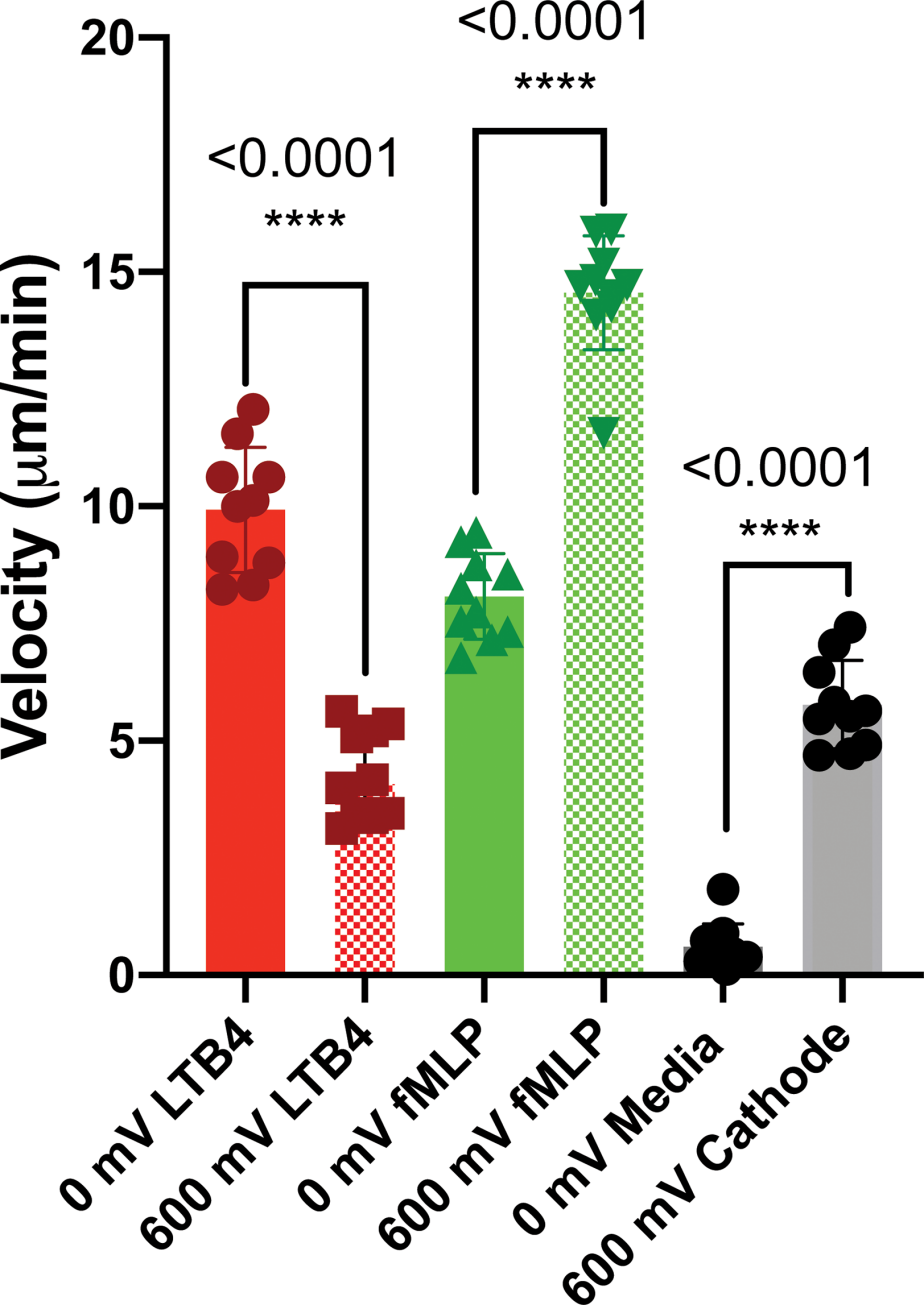
dHL60 single cell velocity under the influence of electrochemical gradient (n=10). The Electrotaxis-On-Chip (ETOC) microfluidic platform enabled us to quantify single-cell neutrophil-like cells electrotaxis velocity (7.9 µm/min ± 3.6). (ns P > 0.05; *P ≤ 0.05; **P ≤ 0.01; ***P ≤ 0.001; ****P ≤ 0.0001).

The third scenario investigated the potential of electrotaxis to increase neutrophil-like cells directional movement to the cite of infection. fMLP [10 nM] was added to the cathode chamber of the device, (side A) for generating a parallel chemotactic gradient to the electric field to test the third hypothesis. Applying the electric field enhanced the migration of neutrophils toward fMLP chemoattractant significantly. In some cases, such as 80 mV (n=4, p-value<0.001) and 600mV (n=4, p-value<0.001) the effect of the electric potential was more significant. In other cases, such as 40 mV (n=4, p-value<0.005), 200 mV (n=4, p-value<0.005), and 400 mV (n=4, p-value<0.005) the significant enhancement in migration was observed (**Figure 5A** and **Supplementary Video 4**). As expected, according to the first two scenarios, the migration of neutrophils toward the anode is 86% (600mV), 62% (400 mV), 60% (200 mV), 90% (80 mV), and 42% (40 mV) less than the cathode (**Figure 5A**). Sides of the device with the complete media demonstrated no significant migration due to the elimination of electrochemical gradients (**Figure 5**). The electric potential spectrum for the third scenario, parallel and synergistic fMLP and electrotaxis, has been shown in **Supplementary Figure 4**. The neutrophil-like single cells velocity under the influence of electrochemical gradient was investigated. The Electrotaxis-On-Chip (ETOC) microfluidic platform enabled us to quantify single-cell neutrophil-like cells electrotaxis velocity in three scenarios: 1. 600 mV electric potential without a chemical gradient (7.9 µm/min ± 3.6). 2. Competing 600mV electric potential and 10nM LTB4 chemoattractant gradient (2.9 µm/min ± 1.7). 3. Synergistic 600mV electric potential and 10nM fMLP chemoattractant gradient (14.8 µm/min ± 2.6) (**Figure 6**).

## Discussion

Electrical stimuli are known to manipulate cells, providing potential therapeutic approaches in treatments of inflammatory diseases. The electrotaxis-on-chip (ETOC) platform developed here will give immunologists a platform for investigating the physiological roles and mechanisms of electrotaxis in a more efficient way and to optimize treatment parameters *in vitro* before testing in patients or mouse models. Our reductionist approach studies show that: 1) Neutrophil-like cells migrate toward the cathode of a DC electric field; 2) Perpendicular electric fields reduce neutrophil-like cell migration towards an inflammatory chemoattractant (LTB_4_); and 3) Concurrent or parallel electric fields can synergistically increase neutrophil chemotaxis towards an infection (fMLP).

Electrotaxis represents an additional mechanism for the control of leukocyte migration. It is likely to play a role in sites of epithelial injury, and may permit novel approaches for manipulating the positioning of neutrophils and other immune cells to enhance pathogen-killing and vaccine or antitumor responses. Although there is currently no clinical practice for inflammation or infection by directly manipulating electrotaxis of immune cells, electrical treatments for chronic wound with therapeutic benefits have been commonly used by medical practitioners, such as physical therapists. There are even human trials to treating human spinal cord by implanting an oscillating EF stimulator (95). The potential electrotaxis-based therapeutic approach for infectious disease or reducing inflammation is likely safely and cost efficiently because the EF is applied at low magnitude using relatively simple electrical setups. On the other hand, it will be critical to optimize the applied EF in clinical applications using enabling platforms such as the ETOC developed here. These platforms can also be used to better understand the molecular mechanisms driving immune cell electrotaxis. A better understanding of EF guided immune cell migration will inspire the development of new EF-based treatments or other biophysical energies that can modulate physiological EF for inflammatory disorders, immunotherapies or other clinical applications. In future, further advances in the design of high-throughput microfluidic devices, more neutrophil chemoattractant (e.g. IL-8) investigation, and using isolated primary neutrophils from patient samples are recommended. The design of microfluidic device can be improved by pressure vapor deposition of the electrodes on glass surface instead of manually inserting the electrodes inside the PDMS device. Also, the location of the cell loading reservoir can be fixed with high accuracy to improve the distribution of electric current lines in the chip.

## Data Availability Statement

The raw data supporting the conclusions of this article will be made available by the authors, without undue reservation.

## Author Contributions

MM ran the experiments. CJ and MM wrote the manuscript. All authors contributed to the article and approved the submitted version.

## Funding

CJ acknowledge funding from The National Institute of General Medical Sciences of the National Institutes of Health under award number R35GM133610.

## Conflict of Interest

The authors declare that the research was conducted in the absence of any commercial or financial relationships that could be construed as a potential conflict of interest.

## Publisher’s Note

All claims expressed in this article are solely those of the authors and do not necessarily represent those of their affiliated organizations, or those of the publisher, the editors and the reviewers. Any product that may be evaluated in this article, or claim that may be made by its manufacturer, is not guaranteed or endorsed by the publisher.
