# Supplementary material for: Electrotaxis-on-Chip to Quantify Neutrophil Migration Towards Electrochemical Gradients
Source: Front Immunol. 2021 Aug 6;12:674727. doi: 10.3389/fimmu.2021.674727 (PMC8379007; doi:10.3389/fimmu.2021.674727)
Supplement: Supplementary file 1 [file DataSheet_1.docx]

Supplementary Material

Electrotaxis-on-chip to quantify neutrophil migration towards electrochemical gradients

Maryam Moarefian, , Rafael V. Davalos, Michael D. Burton, Caroline N. Jones*

*** Correspondence:** Corresponding Author: CNJ (email: [Caroline.Jones@utdallas.edu](mailto:Caroline.Jones@utdallas.edu))

**Supplementary Figure 1.** **Viability of dHL60s after applying 600 mV electric field for 8h. (A)** Live/dead image at t= 6 min. Stained dHL60s by live and dead cells fluorescent assay (Abcam, Cambridge, MA) illustrated high percent viability at the beginning of the experiment (t= 6 min). **(B)** Live/dead image at t= 8h under the effect of an electrochemical gradient. Stained dHL60s under the effect of two conditions: 10nM of LTB_4_ chemoattractant and the 600-mV electric potential demonstrated the high percentage of viability. **(C)** Live/dead image at t= 8h under the effect of the electric field. Stained dHL60s under the effect 600-mV electric potential indicated the high percentage of viability. **(D)** Zoomed image of Figure 1S.C. **(E)** The quantification of percent viability of dHL60s under three different conditions. dHL60 viability in the microfluidic device (n = 2) proved after 8 hours of migration experiment, dHL60 cells loaded into the microfluidic platform were >90% viable.

**
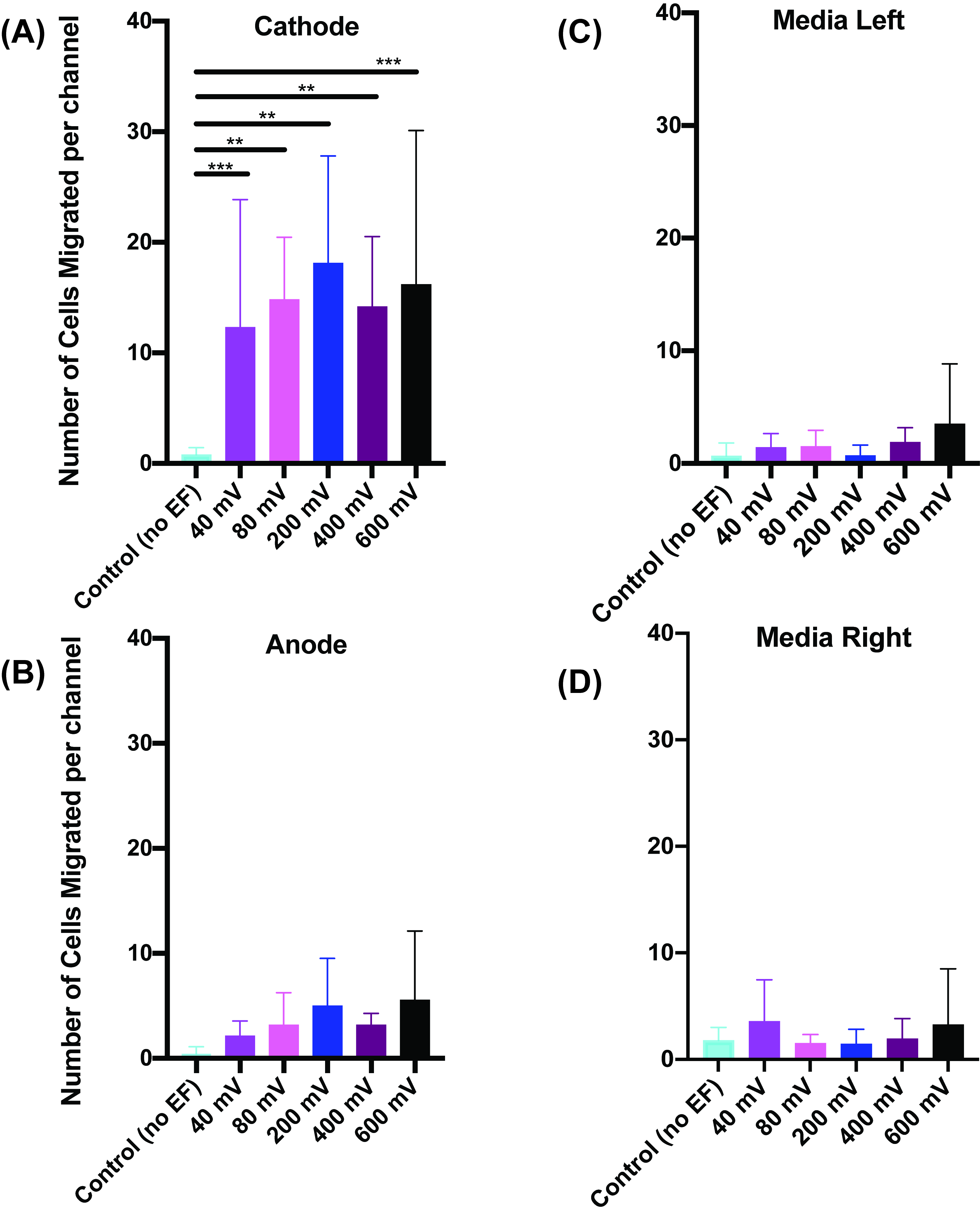
**

**Supplementary Figure 2**. **Electric potential spectrum for number cells migrated per channel under the influence of electrotaxis only.** **(A)** Number of cells migrated toward cathode (n=4, p-value<0.005). **(B)** Number of cells migrated toward Anode. **(C)** Number of cells migrated toward media on the left side of the device. **(D)** Number of cells migrated toward the media on right of the device.

**
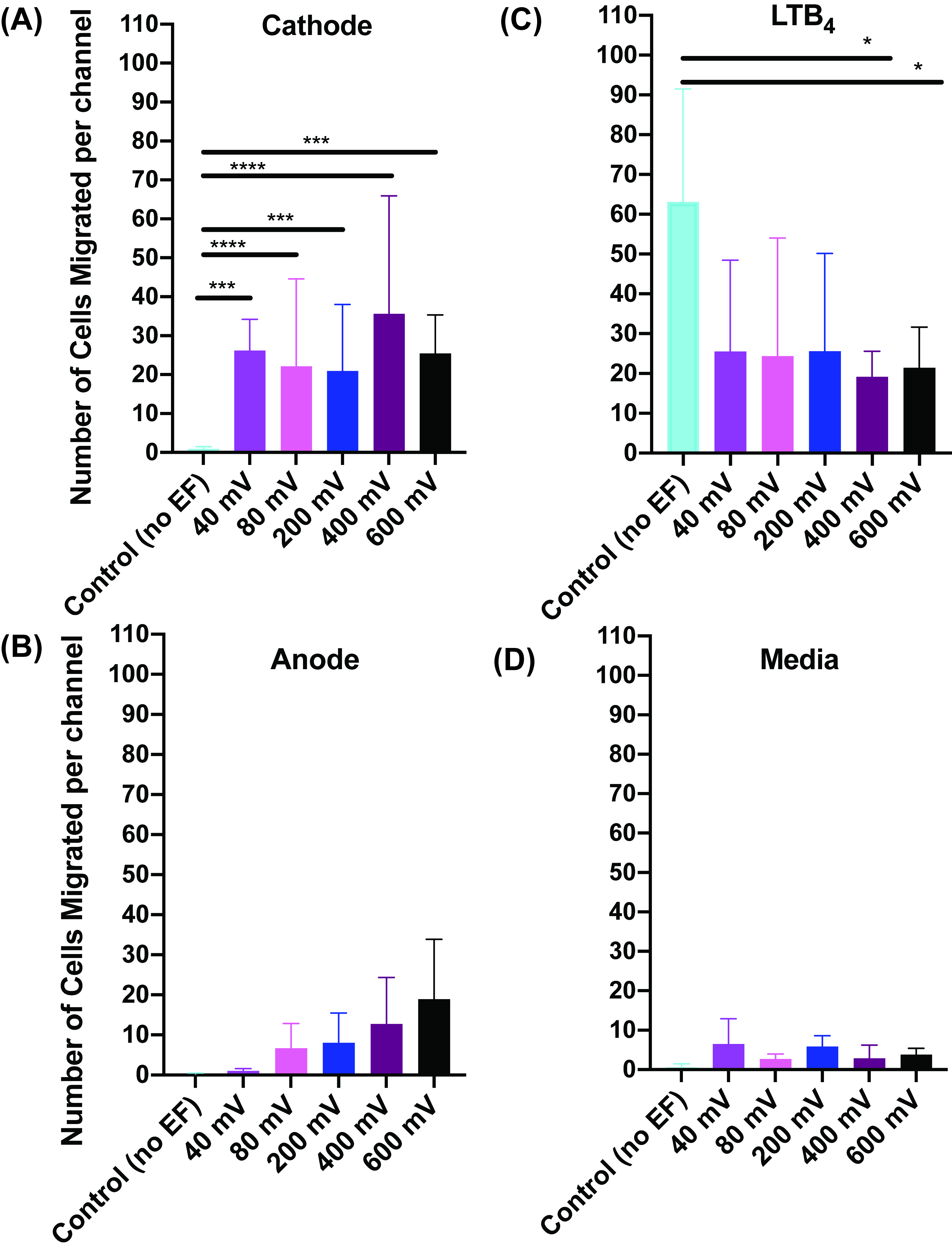
**

**Supplementary Figure 3. Electric potential spectrum for number cells migrated per channel under the influence of perpendicular and competing LTB4 and electrotaxis. (A)** Number of cells migrated toward cathode (n=4, p-value<0.005). **(B)** Number of cells migrated toward Anode. **(C)** Number of cells migrated toward 10nM LTB_4_ (n=4, p-value<0.005). **(D)** Number of cells migrated toward the media.

**
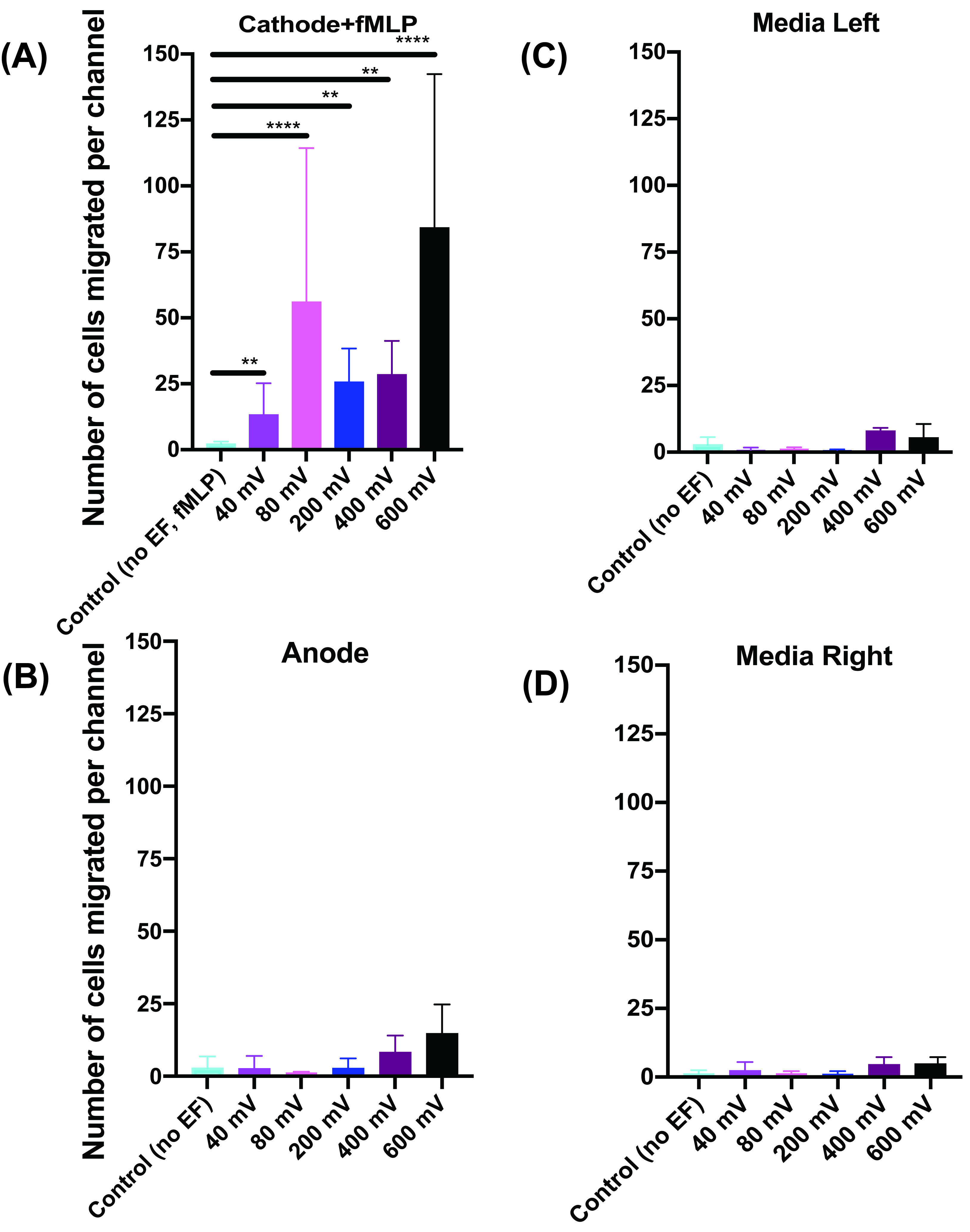
**

**Supplementary Figure 4. Electric potential spectrum for number cells migrated per channel under the influence of parallel and synergistic fMLP and electrotaxis. (A)** Number of cells migrated toward cathode and 10nM fMLP (n=4, p-value<0.005). **(B)** Number of cells migrated toward Anode. **(C)** Number of cells migrated toward media on the left side of the device. **(D)** Number of cells migrated toward the media on right of the device.

**Supplementary Videos**

**Supplementary Video 1.** **Electrotaxis of dHL60 cells toward cathode within the migration channel.** dHL60 cell migration within ETOC (Electrotaxis-On-Chip) device under the influence of 600mV electric field. Time 8h, Scale bar 100µm

**Supplementary Video 2. dHL60 cells do not migrate toward anode within the migration channel.** dHL60 cells toward the anode of -200mV electric potential. Time 8h, Scale bar 100µm

**Supplementary Video 3. Competitive and perpendicular electrotaxis and chemotaxis of dHL60 cells.** dHL60 cells migrate toward the cathode of 600mV electric potential even in the presence of a competitive LTB_4_ [100 nM] gradient. Time 8h, Scale bar 100µm

**Supplementary Video 4.** **Parallel electrotaxis and chemotaxis of dHL60 cells.** dHL60 cell migration toward 600mV electric potential, which is parallel to fMLP [10 nM] gradient. Electric potential increased migration of dHL60s toward fMLP gradient. Time 8h, Scale bar 100µm.
